# Durable cross-protective neutralizing antibody responses elicited by lipid nanoparticle-formulated SARS-CoV-2 mRNA vaccines

**DOI:** 10.1038/s41541-024-00835-x

**Published:** 2024-02-23

**Authors:** Ki Hyun Bae, Bhuvaneshwari Shunmuganathan, Li Zhang, Andrew Lim, Rashi Gupta, Yanming Wang, Boon Lin Chua, Yang Wang, Yue Gu, Xinlei Qian, Isabelle Siang Ling Tan, Kiren Purushotorman, Paul A. MacAry, Kevin P. White, Yi Yan Yang

**Affiliations:** 1 Bioprocessing Technology Institute (BTI), Agency for Science, Technology and Research (A*STAR), 20 Biopolis Way, Centros #06-01, Singapore, 138668 Republic of Singapore; 2 Department of Microbiology and Immunology, Yong Loo Lin School of Medicine, National University of Singapore, Singapore, 117545 Republic of Singapore; 3 NUS-Cambridge Immune Phenotyping Centre (NCIPC), Life Sciences Institute, National University of Singapore, Singapore, 117456 Republic of Singapore; 4 Provaxus, Inc, Dover, Delaware 19901 USA; 5 Genome Institute of Singapore (GIS), Agency for Science, Technology and Research (A*STAR), 60 Biopolis St, Singapore, 138672 Republic of Singapore; 6 Department of Biochemistry and Precision Medicine Translational Research Program, Yong Loo Lin School of Medicine, National University of Singapore, Singapore, 119228 Republic of Singapore

**Keywords:** RNA vaccines, RNA vaccines

## Abstract

The advent of SARS-CoV-2 variants with defined mutations that augment pathogenicity and/or increase immune evasiveness continues to stimulate global efforts to improve vaccine formulation and efficacy. The extraordinary advantages of lipid nanoparticles (LNPs), including versatile design, scalability, and reproducibility, make them ideal candidates for developing next-generation mRNA vaccines against circulating SARS-CoV-2 variants. Here, we assess the efficacy of LNP-encapsulated mRNA booster vaccines encoding the spike protein of SARS-CoV-2 for variants of concern (Delta, Omicron) and using a predecessor (YN2016C isolated from bats) strain spike protein to elicit durable cross-protective neutralizing antibody responses. The mRNA-LNP vaccines have desirable physicochemical characteristics, such as small size (~78 nm), low polydispersity index (<0.13), and high encapsulation efficiency (>90%). We employ in vivo bioluminescence imaging to illustrate the capacity of our LNPs to induce robust mRNA expression in secondary lymphoid organs. In a BALB/c mouse model, a three-dose subcutaneous immunization of mRNA-LNPs vaccines achieved remarkably high levels of cross-neutralization against the Omicron B1.1.529 and BA.2 variants for extended periods of time (28 weeks) with good safety profiles for all constructs when used in a booster regime, including the YN2016C bat virus sequences. These findings have important implications for the design of mRNA-LNP vaccines that aim to trigger durable cross-protective immunity against the current and newly emerging variants.

## Introduction

The outbreak of the COVID-19 pandemic in 2019, stimulated tremendous global efforts to develop messenger RNA (mRNA) based vaccines aimed at preventing infection and/or reducing disease severity following exposure to SARS-CoV-2. The mRNA-1273 and BNT162b2 vaccines from Moderna and BioNTech/Pfizer, received the U.S. Food and Drug Administration (FDA) approval based upon their efficacy (~90%) against the ancestral Wuhan (Wuhan-Hu-1) variant of SARS-CoV-2^1,2^. The success of these two vaccines has been built upon the use of nucleoside-modified mRNA-loaded lipid nanoparticles (mRNA-LNPs). The pseudouridine modification improves the biological stability and translational efficiency of mRNA, while attenuating its immunogenicity in vivo^3,4^. The distinctive advantages of LNP-based mRNA delivery systems, such as low-cost manufacturing, versatile design, scalability, and reproducibility, have contributed to the advancement of SARS-CoV-2 vaccines at an unprecedented pace and scale^5^. Moreover, encapsulation of the mRNA in LNPs effectively protects it from nuclease-mediated degradation and enables its cytosolic delivery by triggering endosomal escape via a pH-dependent membrane-destabilizing property of an ionizable lipid containing a tertiary amine group^6^. Following intramuscular vaccination, LNPs are designed to translate mRNA encoding the SARS-CoV-2 spike glycoprotein for extended periods of time, resulting in humoral antibody responses and protective immunity in humans^7^.

While the existing mRNA vaccines have been proven effective against the Wuhan-Hu-1 strain, the evolution of the spike protein has led to the emergence of diverse variants of SARS-CoV-2 with greater infectivity and immune-evasive capacity^8^. For instance, the Delta variant (B.1.617.2), which was first identified in late 2020 and spread globally to 135 countries, possesses greater transmissibility and results in poorer vaccine efficacy than the Wuhan-Hu-1 strain through the enhanced affinity of the mutated spike towards angiotensin-converting enzyme 2 (ACE2)^9,10^. The Omicron variant (B.1.1.529) and its sublineages carry a larger number of mutations in the Spike receptor-binding domain (RBD) than the Delta variant, and rapidly became the dominant variant in most countries by mid-2022^11^. These SARS-CoV-2 variants are highly resistant to mRNA vaccine-induced humoral immunity; two doses of mRNA-1273 or BNT162b2 vaccines achieved only 28–44% and 68% of protective efficiencies against the Delta and Omicron variant, respectively^12^. Recently, bivalent boosters containing mRNA directed against both ancestral strain and Omicron BA.1 have been developed, but they induced only 1.5 to 1.75-fold higher levels of virus-neutralizing antibodies compared to monovalent boosters^13^. In addition, the use of bivalent mRNA boosters targeting both the ancestral strain and BA.4–BA.5 variant failed to promote a better neutralizing antibody response against Omicron sublineages over the original monovalent vaccine booster^14^. Collectively, these highlight an urgent need for more effective vaccination strategies for SARS-CoV-2 variants aimed at stimulating broader and more durable immune responses.

**Fig. 1:**
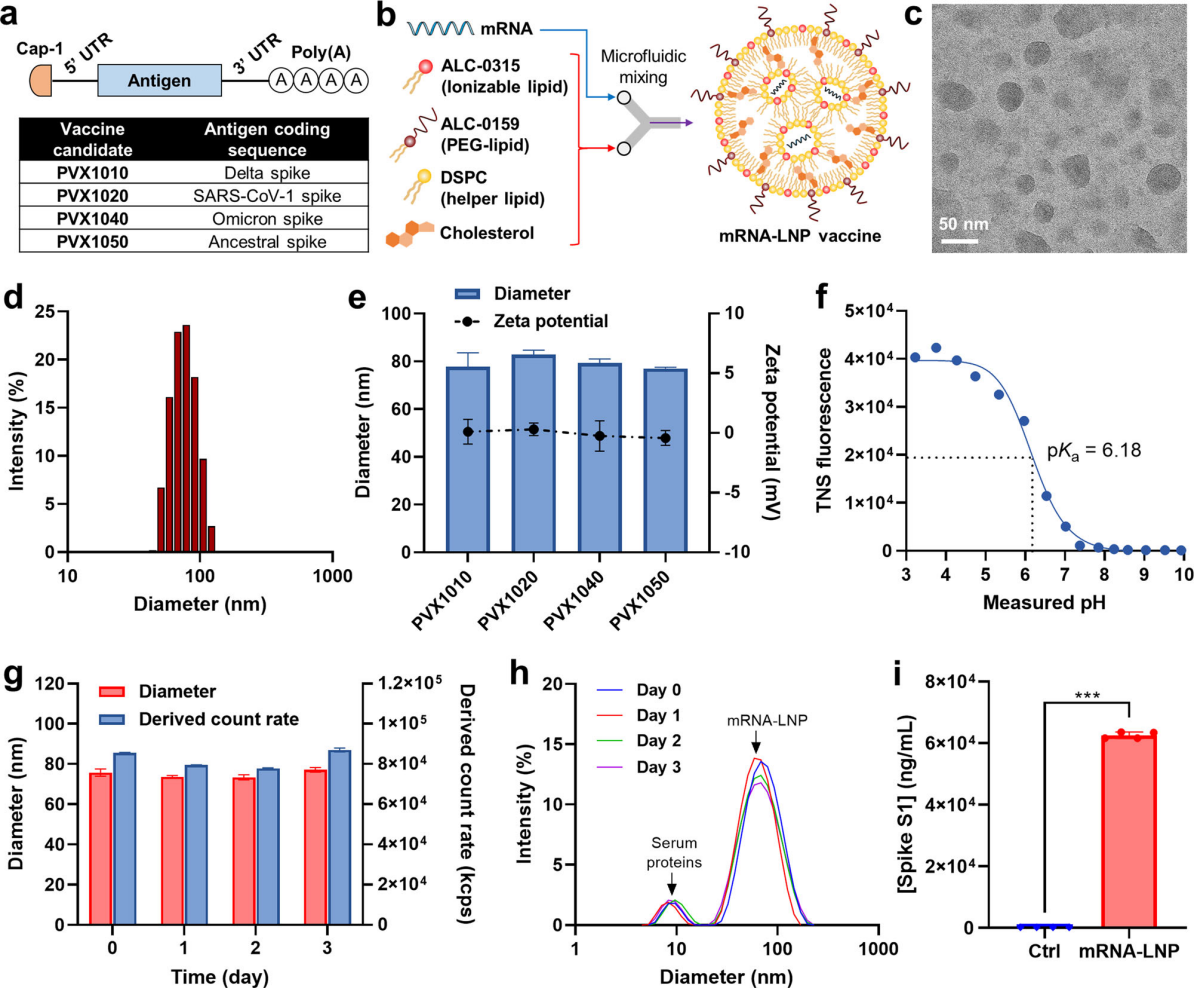
Design of lipid nanoparticle (LNP)-encapsulated mRNA vaccines targeting SARS-CoV-2 variants. **a** Linear diagram of the structural elements of mRNAs encoding the spike glycoprotein of SARS-CoV-2 variants. **b** Schematic view on production of mRNA-LNP vaccine using a microfluidic device. **c** Representative cryo-TEM image of mRNA-LNPs. The scale bar represents 50 nm. **d** Size distribution profile of mRNA-LNPs. **e** Comparison of the hydrodynamic diameter and zeta potential values of PVX1010 (Delta), PVX1020 (SARS-CoV-1), PVX1040 (Omicron) and PVX1050 (YN2016C) mRNA-LNPs. **f** Surface ionization property of mRNA-LNPs measured by TNS binding assay. The acid dissociation constant (p*K*_a_) was determined to be 6.18. **g** The diameter and derived count rate of mRNA-LNPs in 10% serum-supplemented PBS as a function of time. **h** Size distribution profiles of mRNA-LNPs incubated in 10% serum-supplemented PBS over 3 days. **i** Concentration of Delta variant spike S1 proteins in the supernatant of HEK293 cells treated with PVX1010 mRNA-LNPs for 2 days. Mean ± SD (*n* = 3); ^***^P < 0.001 (statistical analysis was performed using two-tailed unpaired Student’s *t* tests).

In this study, we developed and tested mRNA-LNP vaccines (PVX1010, PVX1020, PVX1040, and PVX1050; Fig. 1a) targeting SARS-CoV-2 variants, and we evaluated their vaccination efficacy and safety in a mouse model. We designed the codon-optimized mRNA PVX1010 and PVX1040, encoding the full-length spike glycoprotein of the Delta and Omicron variants, respectively. To test the cross-immunity potential to more distantly related coronavirus strains, we also designed PVX1020 mRNA for the SARS-CoV-1 spike glycoprotein and PVX1050 mRNA for YN2016C, which has been reported by others to be a potential direct ancestor to SARS-CoV-2^15^. All the mRNAs were modified to include two consecutive proline substitutions in the S2 subunit (Supplementary Fig. 1) to stabilize the spike protein in its antigenically optimal perfusion conformation^16^. mRNA-LNPs were formulated by microfluidic mixing of lipids that have been used for Pfizer-BioNTech’s COVID-19 mRNA vaccine^1^. Physicochemical characterization was conducted to understand the nanoparticle structure, surface charge property, and physiological stability of the produced mRNA-LNPs. In vivo, bioluminescence imaging was undertaken to assess the mRNA expression patterns of mRNA-LNPs in different organs. We evaluated the effectiveness of a three-dose subcutaneous immunization regimen in a BALB/c mouse model. Interestingly, two doses of the Delta variant vaccine PVX1010 plus either a homologous or heterologous booster resulted in robust, cross-protective neutralizing antibody responses against the Omicron B1.1.529 and BA.2 variants for over 28 weeks without severe toxicity. This study may provide valuable information that sparks the advancement of mRNA-LNP vaccines conferring durable cross-protection against emerging variants of SARS-CoV-2.

## Results

### Preparation and characterization of SARS-CoV-2 spike mRNA-LNPs

mRNA-LNPs were formulated in a microfluidic device by controlled mixing of mRNA with an ethanolic solution of ALC-0315 (an ionizable lipid), ALC-0159 (a polyethylene glycol (PEG)-lipid conjugate), DSPC (a zwitterionic helper lipid) and cholesterol (Fig. 1b). The lipid compositions were selected based on the conventional LNP formulation used for Pfizer-BioNTech mRNA vaccine^1^. During the microfluidic mixing process, the electrostatic interactions between the anionic mRNA and ionizable amine group of ALC-0315 lead to the encapsulation of mRNA in a lipid shell covered by PEG-linked lipid, which provides a steric barrier to avoid aggregation during particle formation^17^. Cholesterol and DSPC are mainly enriched at the surface of LNP and known to contribute to its structural stability^18^. The formation of well-dispersed spherical mRNA-LNPs was confirmed by cryogenic TEM (Fig. 1c). Dynamic light scattering (DLS) elucidated that mRNA-LNPs had a monodisperse size distribution of 77.9 ± 5.8 nm with polydispersity index of <0.13 (Fig. 1d). The hydrodynamic diameter, zeta potential values (close to neutral) and encapsulation efficiency (>90%) remained consistent over all the tested mRNAs, highlighting the versatility of the LNP formulation (Fig. 1e and Supplementary Table 1). Using TNS fluorescence measurements, the apparent p*K*_a_ of mRNA-LNP was determined to be 6.18 (Fig. 1f), which was similar to that of ALC-0315 (6.09) reported in the literature^19^. mRNA-LNPs did not show notable changes in the hydrodynamic diameter and derived count rate in 10% serum-supplemented phosphate-buffered saline (PBS) over 3 days (Fig. 1g), indicating their high colloidal stability in the physiological environment. As shown in Fig. 1h, mRNA-LNPs were able to maintain their size distribution in the presence of serum proteins, which appeared as a small peak of ~8.3 nm (Supplementary Fig. 2). Abundant expression of SARS-CoV-2 spike S1 proteins (~62,600 ng/mL) was observed in the supernatant of HEK293 cells treated with mRNA-LNPs for 2 days (Fig. 1i), reflecting the S protein cleavage via cellular proteases^20^.

### In vivo and ex vivo bioluminescence imaging study

**Fig. 2:**
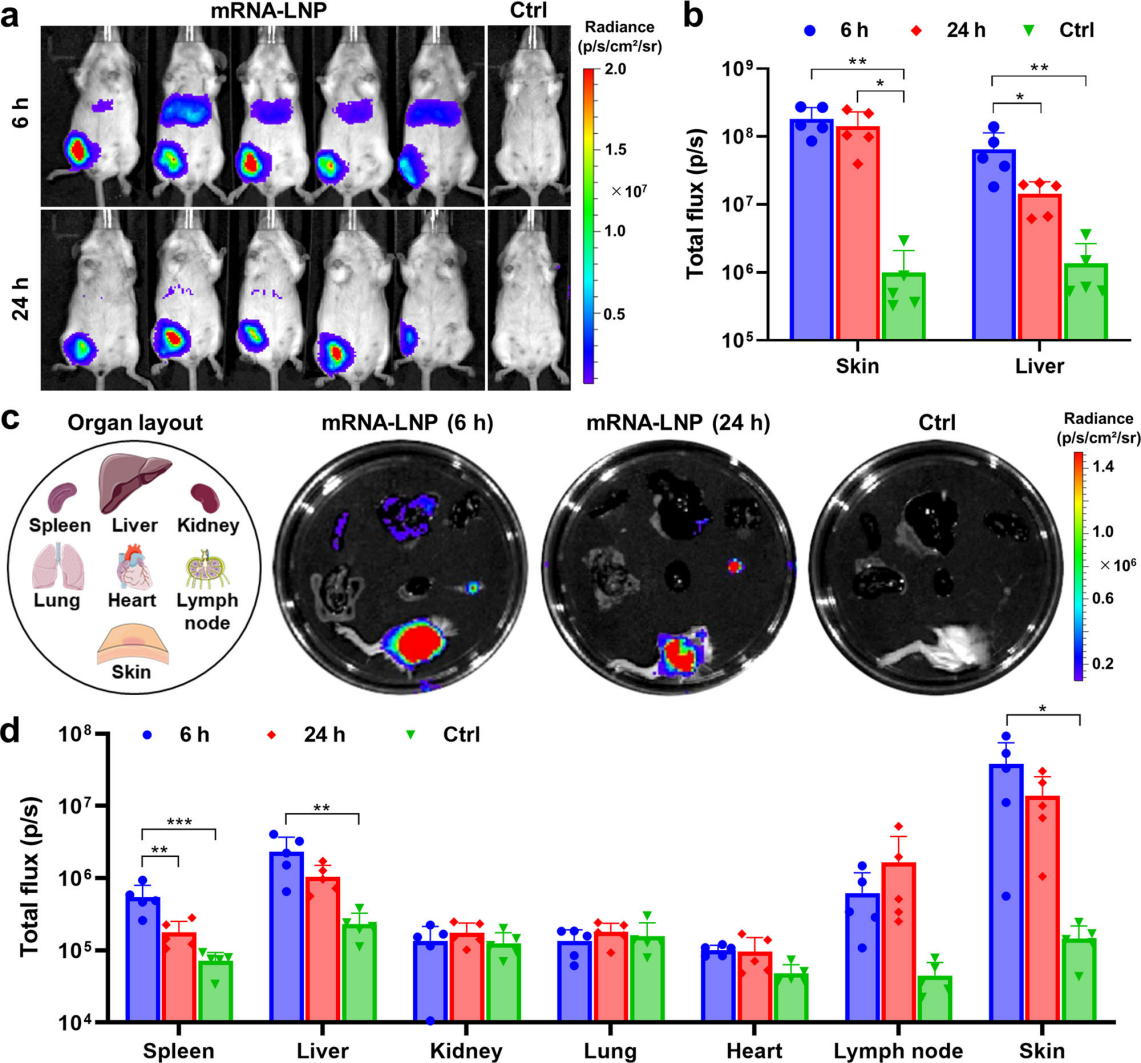
Assessment of in vivo translation profiles of FLuc reporter-encoding mRNA-LNPs in BALB/c mice. **a** In vivo bioluminescence images of BALB/c mice taken at 6 and 24 h following subcutaneous administration of FLuc mRNA-LNPs. Naive mice injected with luciferin served as a mock control (Ctrl). **b** Total flux values of skin (injection site) and liver areas measured at 6 h and 24 h post-administration. Mean ± SD (*n* = 5). **c** Schematic organ layout and representative ex vivo luminescence images of major organs dissected from the mice. **d** Quantification of total flux values of the collected organs. Mean ± SD (*n* = 5); ^*^P < 0.05; ^**^P < 0.01; ^***^P < 0.001 (statistical analysis was performed using one-way ANOVA with Tukey’s post hoc test).

Next, we verified the biodistribution of mRNA-LNPs using in vivo bioluminescence imaging. To visualize the in vivo translation profiles, a firefly luciferase (FLuc) reporter-encoding mRNA-LNPs were formulated using the same procedure as SARS-CoV-2 vaccine mRNA-LNPs and administered to BALB/c mice *via* subcutaneous route. At 6 h post administration, robust FLuc expression was localized in the skin (injection site) as well as in the liver (Fig. 2a). At 24 h, the bioluminescence signals were predominantly confined to the skin, whereas those in the liver faded to almost undetectable levels. This finding was corroborated by the observation that the total flux remained constantly high in the skin over 24 h despite its drastic decline in the liver (Fig. 2b). To further examine the translation pattern of mRNA-LNPs, major organs were dissected from the mice and subjected to ex vivo luminescence imaging (Fig. 2c). FLuc expression was the most pronounced in the skin, with some expression observed from the lymph node, liver and spleen at 6 h post-administration (Fig. 2d). Notably, FLuc signal of the lymph node, liver and skin at 24 h were comparable to those observed at 6 h, suggesting that the mRNA expression in these organs persisted for at least 24 h. The enhanced lymph node delivery of mRNA-LNP would be advantageous because the lymph node is a primary site for the initiation of adaptive immune responses^21^. For the kidney, lung, and heart, the luminescence signals of the mRNA-LNPs-treated group were similar to those of the mock control group, indicative of negligible mRNA expression in those organs.

### Immunogenicity of SARS-CoV-2 mRNA-LNP vaccines in BALB/c mice

**Fig. 3:**
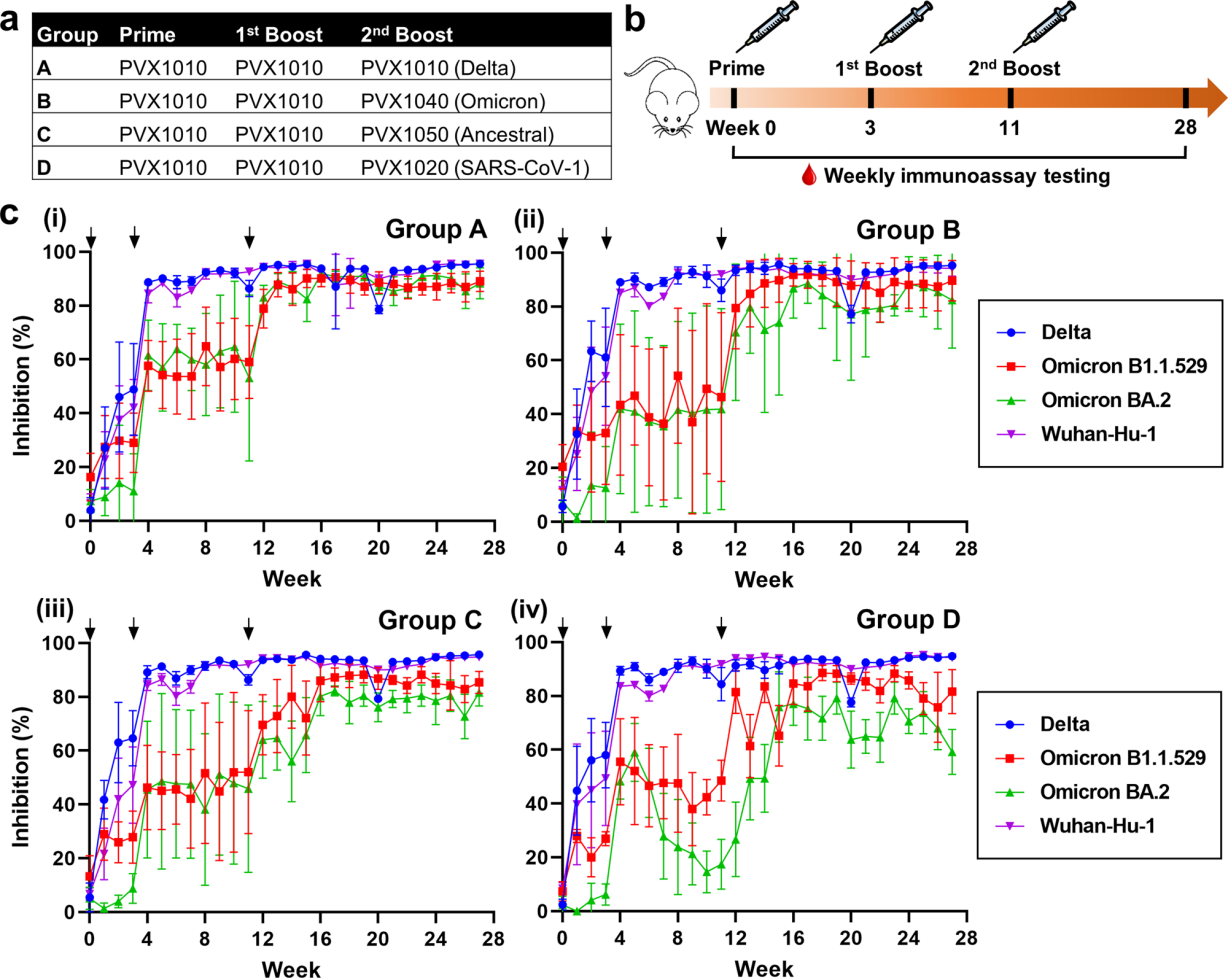
Induction of neutralizing antibody responses in BALB/c mice immunized with 4 different vaccination schedules. **a** Table showing the list of mRNA-LNPs used for the prime, 1st boost and 2nd boost immunization. **b** Schematic diagram of prime-boost immunization and immunoassay schedule. **c** ACE2-RBD binding inhibitory response in serum of the vaccinated BALB/c mice over 28 weeks for 4 different vaccination groups; (i) Group A (a three-dose immunization of PVX1010 mRNA-LNPs), (ii) Group B (two doses of PVX1010 plus PVX1040 booster), (iii) Group C (two doses of PVX1010 plus PVX1050 booster), (iv) Group D (two doses of PVX1010 plus PVX1020 booster). The arrows depict the time points of immunization (weeks 0, 3, and 11). Mean ± SD (*n* = 5).

A human ACE2-Spike RBD binding inhibition ELISA, was used as a surrogate viral neutralization test^22,23^, to measure the percentage of neutralizing antibodies present in vaccinated serum samples, which serves as an indicator of cross-protective immunity against the various variants of SARS-CoV-2. This assay was utilized to evaluate the strength of the humoral response elicited post administration of different mRNA vaccine construct formulations in mice. The mice were separated into four different groups and administered the respective vaccine formulations using the prime, 1st boost, 2nd boost regime as indicated (Fig. 3a). The mRNA vaccines were administered at various time points as illustrated in Fig. 3b, and blood was collected weekly and processed to obtain the serum. We observed a significant rise in the level of percentage ACE2 inhibition against only the Delta and Wuhan-Hu-1 variants across all groups from week 0 (prime vaccine dose using Delta mRNA-LNPs) to week 3 when the 1st boost using Delta mRNA-LNPs was administered. The percentage inhibition levels remained below the threshold of 40% against the Omicrons B1.1.529 and BA.2 variants. From week 3 to week 11 prior to the 2nd boost, inhibition levels remained above 80% at a steady plateau for the Delta and Wuhan-Hu-1 variants. This trend was observed continually post-2nd boost using Delta, Omicron, YN2016C, or SARS-CoV-1 mRNA-LNPs from week 11 to week 28. In contrast, only a slight increase in percentage ACE2 inhibition levels was observed from week 3 to week 11 against the Omicron variants. Although unexpectedly broad neutralizing antibody responses ranging from 20% to 60% were observed for the Omicron 1.1.529 and BA.2 variants from week 4 to week 10, there were no significant (*P* > 0.05) differences among all the vaccination groups during the period of study (Supplementary Fig. 3). However, the ACE2-RBD inhibitory responses to the Delta and Wuhan-Hu-1 variants were significantly higher than those to the Omicrons B1.1.529 and BA.2 variants (Supplementary Fig. 4). After week 11, the ACE2 inhibition levels against the Omicron variants showed a substantial increase comparable to that of the Delta and Wuhan-Hu-1 strains across all groups post-2nd boost. These data indicate that a 2nd boost with each of the vaccine formulations increases the cross-protective immunity levels, especially for the Omicron variants.

### Safety profiles of SARS-CoV-2 mRNA-LNP vaccines

**Fig. 4:**
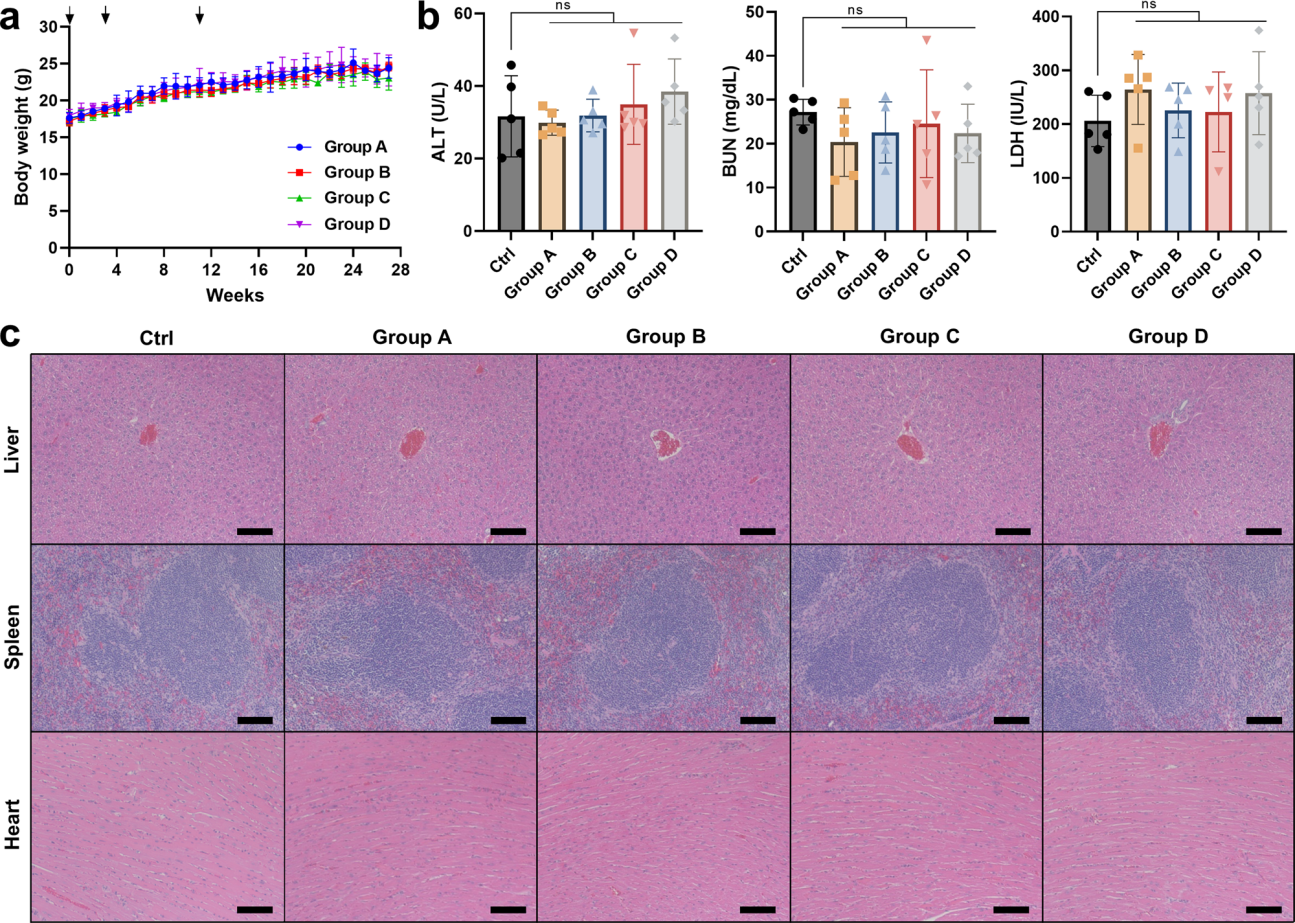
Evaluation of tolerability of SARS-CoV-2 mRNA-LNP vaccines in BALB/c mice. **a** Body weight changes of BALB/c mice in the course of vaccination study. The arrows depict the time points of immunization (week 0, 3, and 11). **b** Serum concentration of ALT, BUN, and LDH detected at the endpoint (week 28). Mean ± SD (*n* = 5); ns: nonsignificant (statistical analysis was performed using one-way ANOVA with Tukey’s post hoc test). **c** Representative H&E-stained sections of the liver, spleen, and heart were harvested at the endpoint. The scale bars represent 100 µm.

No significant weight loss was detected from all the groups throughout the course of the vaccination study (Fig. 4a). Blood chemistry analysis was also undertaken to measure the serum concentration of blood urea nitrogen (BUN), alanine transaminase (ALT), lactate dehydrogenase (LDH), which are well-known markers of kidney, liver and heart dysfunction. As shown in Fig. 4b, all the mice treated with mRNA-LNPs had comparable serum levels of ALT, BUN and LDH to the untreated control mice. Moreover, histological analysis of the liver, spleen, and heart did not reveal any sign of necrosis, inflammation, or other abnormal morphological characteristics from all the vaccinated mice (Fig. 4c). Collectively, the above results demonstrated the cross-protective immunization efficacy and tolerability of SARS-CoV-2 vaccine mRNA-loaded LNPs.

## Discussion

In the present study, mRNA-LNP vaccine was constructed by mixing an aqueous solution of mRNA with lipids dissolved in ethanol *via* a microfluidic device. The physicochemical characterization of mRNA-LNPs confirmed the successful production of monodisperse spherical mRNA-LNPs with an average hydrodynamic diameter of ~78 nm and mRNA encapsulation efficiency greater than 90%. It has been reported that the extracellular pH remains constant at ~7.4, while the matured endosomes create an acidic environment (pH ~5.5)^24^. The apparent p*K*_a_ of mRNA-LNP was determined to be 6.18 by TNS binding assay. This finding suggests that mRNA-LNPs may have a near-neutral surface charge during blood circulation, but protonate to form a cationic surface once internalized by cells and enter endosomes in the cells, thus facilitating its endosomal disruption and cytosolic delivery of mRNA cargo^19^. Colloidal stability is another important factor influencing the performance of mRNA-LNP vaccines administered in vivo. Subcutaneous administration typically results in the delivery of compounds to the interstitial area underneath the dermis before they reach systemic circulation via local vasculature or draining lymphatics^25,26^. It has been reported that the interstitial tissue fluid contains similar protein components to plasma, although its total protein content is about 68% lower than that of plasma^27^. In this regard, the physiological stability of mRNA-LNP vaccine was assessed in 10% serum-supplemented PBS. When incubated with 10% serum over 3 days, mRNA-LNPs showed negligible changes in the hydrodynamic diameter, derived count rate, and particle size distribution, demonstrating its good colloidal stability in the physiological environment. This phenomenon was likely attributed to the existence of PEG-lipid conjugate, which provides a steric shield layer to avoid nonspecific adsorption of serum proteins onto mRNA-LNPs^28^.

To date, intramuscular injection has been chosen as the route of vaccination for both the BNT162b2 and mRNA-1273 vaccines as well as those under clinical trials. However, a recent study has discovered that subcutaneous immunization of BNT162b2 vaccine resulted in less severe adverse events than intramuscular immunization, without compromising the humoral immune response^29^. Based on this perspective, subcutaneous route was selected to investigate the in vivo biodistribution profile and vaccination efficacy of mRNA-LNPs in our study. The bioluminescence imaging study revealed that FLuc mRNA expression following subcutaneous injection was largely detected in the site of injection, lymph node, liver, and spleen. Negligible mRNA expression was seen in the kidney, lung and heart, which was in accordance with the biodistribution pattern of mRNA-LNPs containing another ionizable lipid (DLin-MC3-DMA) following subcutaneous administration^30^. The robust mRNA expression in the lymph node and spleen is considered beneficial for vaccination because these lymphoid organs are known to play a central role in mediating vaccine-specific adaptive immunity. Augmented expression of the spike antigen at anatomical sites that represent foci for initiating adaptive immunity is an important parameter when considering the likely in vivo efficacy of our immunization approach.

The surge of diverse SARS-CoV-2 variants has prompted extensive research on the cross-neutralizing humoral immunity of mRNA vaccines. Recently, Lee et al.^31^ designed Delta × Omicron hybrid mRNA vaccine that carries all 16 point-mutations found in the RBD of Delta and Omicron in a single construct and compared its cross-immunization effect with those of Delta- and Omicron-specific mRNA vaccines. The neutralizing antibody response of the Omicron and hybrid mRNA vaccines was only limited to an Omicron variant, whilst the Delta mRNA vaccine produced broad antibody responses against multiple variants. Roth et al.^32^ reported that N1-methylpseudouridine-modified mRNA vaccine (CV0501) directed against Omicron BA.1 exerted a high neutralizing effect on only Omicron, while displaying a marginal response on ancestral, Beta, and Delta variants. Since the prior studies suggest the limited capability of Omicron mRNA vaccination in facilitating cross-protective humoral responses^31,32^, the Omicron spike PVX1040 mRNA was not chosen for prime-boost immunization in our study. In addition, multiple clinical studies reported that BNT162b2 and mRNA-1273 vaccines (targeting the Wuhan-Hu-1 strain) as priming or boosting dose induced a potent neutralizing antibody response against the Wuhan-Hu-1 strain, but not other SARS-CoV-2 variants^33–35^. In this perspective, we attempted to explore the use of the Delta spike PVX1010 mRNA-LNP vaccine as the primer and 1st booster in our immunization regimen.

Our surrogate viral neutralization test revealed that the prime and 1st boost immunization of PVX1010 generated a high level (>80%) of neutralizing antibody responses against Delta and Wuhan-Hu-1 variants in BALB/c mice, whilst modest antibody responses only between 20% and 60% inhibition were observed for Omicron B1.1.529 and BA.2 variants. The substantially reduced neutralization of the Omicron variants was likely caused by the mutational changes in the spike RBD structure, which enabled an escape from vaccine-elicited neutralizing antibodies^34,36^. Notably, the 2nd boost immunization with PVX1010 (Delta) resulted in robust neutralizing antibody responses against the Omicron BA.2 and B1.1.529 variants for over 28 weeks, with a magnitude similar to that of PVX1040 (Omicron). This finding is encouraging because the Omicron spike confers a high resistance to mRNA vaccine-elicited neutralization. For instance, even after 3 doses of BNT162b2 vaccine, Omicron was poorly neutralized by the sera from the vaccinated subjects, with a 53-fold and 23-fold reduction in the neutralization titres over D614G and Delta, respectively^35^. The long duration (~6 months) of persistent neutralizing antibody responses was noteworthy, considering previous clinical studies reporting a gradual decline in mRNA vaccine-induced humoral immunity over a 6–7 month period post-vaccination^37–39^.

Another interesting finding is that PVX1020 (SARS-CoV-1) and PVX1050 (YN2016C) administered as the 2nd booster produced similar neutralizing activities on the Omicron BA.2 and B1.1.529 variants compared to PVX1040 (Omicron), although the Omicron spike shares only 75% and 76% protein sequence identity with the SARS-CoV-1 and YN2016C spike, respectively. These results suggest that the prime-boost immunization with PVX1010, rather than the variant types of the 2nd boost, played a crucial role in mediating the observed cross-variant neutralization of the Omicron variants. The mechanism underlying this durable cross-reactive immunity is unclear but might be associated with the persistent induction of germinal centre responses that recruit cross-reactive memory B cells recognizing epitopes shared among different variants^40,41^. Blood chemistry and histological analysis showed no sign of organ injury and inflammation in the immunized mice, confirming a favorable safety profile of the mRNA vaccines. Taken together, the current study supports the potential of PVX1010, PVX1020, PVX1040, and PVX1050 as promising mRNA vaccine candidates capable of promoting durable cross-protective humoral immunity against diverse variants of SARS-CoV-2.

## Methods

### Materials

Firefly luciferase mRNA (FLuc mRNA) modified with 5-methoxyuridine was supplied by TriLink BioTechnologies (San Diego, CA, USA). ALC-0315, ALC-0159, cholesterol, and 1,2-distearoyl-*sn*-glycero-3-phosphorylcholine (DSPC) were acquired from MedChemExpress (Monmouth Junction, USA). 6-(*p*-Toluidino)-2-naphthalenesulfonic acid sodium salt (TNS) was obtained from Santa Cruz Biotechnology (Santa Cruz, USA). Vivaspin 20 centrifugal concentrator was a product of Sartorius (Goettingen, Germany). Quant-iT RiboGreen RNA assay kit (ThermoFisher, USA), AlamarBlue assay reagent (Life Technologies, USA), and SARS-CoV-2 spike RBD protein sandwich ELISA kit (GeneTex, GTX536267, USA) were used according to the manufacturer’s protocol. HRP-conjugated ACE2-Fc detector antibody used was our in-house antibody. We outsourced it to ThermoFisher (Waltham, MA, USA) for large-scale production (Lot number: 3315928).

### Synthesis of SARS-CoV-2 spike mRNA

All the SARS-CoV-2 spike mRNAs were produced and purified using a proprietary custom process at TriLink BioTechnologies (San Diego, CA, USA). A method for synthesizing mRNA was employed using a single in vitro transcription (IVT) reaction with T7 RNA polymerase and linearized DNA plasmids to create codon-optimized mRNAs (PVX1010, PVX1020, PVX1040, and PVX1050). The reaction utilized TriLink’s CleanCap^®^ reagent, which introduced a modification known as 3’OMe on the m7-Guanosine. Notably, the co-transcriptional capping process achieved an efficiency of over 95% in capping the mRNA. The primary objective of this technique was to enhance translation efficiency within living cells and organisms. The IVT reaction was designed to allow for the complete substitution of regular NTPs with modified Pseudo-UTP instead of UTP. Following the synthesis, the RNA was purified using a spin column-based method, effectively removing unincorporated nucleotides, proteins, and salts. Subsequently, the concentration of the purified mRNA was determined by examining the ultraviolet absorbance at 260 nm, while the molecular weight was determined through gel electrophoresis.

### Production and characterization of mRNA-LNPs

To produce mRNA-LNPs, mRNA was prepared in sodium acetate buffer (1 mM, pH 4.8) to form the aqueous phase. ALC-0315, ALC-0159, DSPC, and cholesterol (molar ratio = 46.3:1.6: 9.4:42.7) were solubilized in ethanol to obtain the organic phase. The aqueous and organic phases (volume ratio = 3:1) were allowed to undergo controlled mixing in the NanoAssemblr Ignite benchtop microfluidic device (Precision Nanosystems, Canada) at a constant flow rate of 12 mL/min. The molar ratio of nitrogen of ALC-0315 to phosphate groups of mRNA (N/P ratio) was fixed to 6:1. The resulting mRNA-LNPs were subjected to a buffer exchange with physiological saline solution and then concentrated using a Vivaspin 20 centrifugal concentrator (*M*_w_ cutoff = 30 kDa). The encapsulation efficiency and total mRNA concentration were determined by the Quant-iT RiboGreen RNA assay kit, as reported previously^42^. Briefly, 100 μL of mRNA-LNPs dispersed in Tris-EDTA buffer (pH 8) was transferred to a black-walled 96-well plate and then mixed with 100 μL of RiboGreen reagent. After incubation for 5 min in a dark place, the fluorescence intensity (FI) was measured on a Spark 10 M microplate reader (Tecan, Switzerland) at 520 nm of emission and at 480 nm of excitation. To quantify the total mRNA concentration, mRNA-LNPs were disrupted by treatment of 5% (v/v) Triton X-100. The encapsulation efficiency was quantified by comparing the ratio of RiboGreen fluorescence before and after treatment of Triton X-100. The diameter and zeta potential values of mRNA-LNPs were examined by DLS analysis using the Zetasizer Ultra Red (Malvern Panalytical, UK). The serum stability of mRNA-LNPs was examined by monitoring their size distribution and derived count rate in 10% serum-supplemented PBS for 3 days.

### Cryo-TEM analysis

Cryo-TEM sample processing and image acquisition were performed at the A*STAR Microscopy Platform (AMP) facility. Briefly, Quantifoil R 2/2 grids (200 mesh copper; EMS, PA, USA) were glow-discharged using the Leica EM SCD005 cool sputter coater (Leica Microsystems, Germany) for 45 seconds at 3–4 mA. Subsequently, 4 μL of mRNA-LNPs was applied to the grids and blotted onto a filter paper for 2 seconds, followed by a snap freezing process in liquid ethane using the GP2 plunge freezer (Leica Microsystems GmbH, Germany). The frozen grid specimens were stored in liquid nitrogen and observed under the Talos L120C transmission electron microscope (FEI, ThermoFisher, USA) operated at 120 kV under the cryo-condition.

### TNS binding assay

The acid dissociation constant (p*K*_a_) of mRNA-LNPs was assessed using the TNS binding assay according to the literature with some modifications^43^. Briefly, a buffer stock solution (150 mM sodium chloride, 10 mM sodium citrate, 10 mM sodium borate, and 10 mM sodium phosphate) was titrated with 0.1 M HCl or NaOH to prepare 15 different buffers with pH values between ~3 and 10. After 90 μL of each buffer was placed in a black-bottomed 96-well plate, 2.6 μL of mRNA-LNPs (mRNA concentration = 50 μg/mL) was added. Subsequently, 2 μL of TNS stock solution (300 μM in DMSO) was topped up to each well. Immediately after repeated pipetting, FI was recorded on Spark 10 M microplate reader (Tecan, Switzerland) at 445 nm of emission and at 321 nm of excitation. The pH of each well was measured by using the Orion ROSS micro pH electrode (ThermoFisher, USA). A scatter plot of TNS fluorescence versus measured pH was created and then fitted to the four-parameter logistic model using the Prism 8 software (GraphPad Software, USA). The apparent p*K*_a_ of mRNA-LNPs was determined as the pH value at which half of the maximum fluorescence was reached.

### Assessment of in vitro mRNA transfection

HEK293 cells (ATCC, USA) were cultivated in DMEM containing 1% (v/v) antibiotics and 10% (v/v) fetal bovine serum (FBS). The cells were seeded on 12-well plates (2 × 10^5^ per well) and incubated in 10% FBS-supplemented media (1 mL) containing PVX1010 mRNA-LNPs with 100 ng of mRNA. After incubation for 2 days, the supernatant was centrifuged at 4 °C for 10 min at 860 × *g*. The concentration of Delta spike S1 proteins in the supernatant was measured by using the SARS-CoV-2 spike RBD protein sandwich ELISA kit (GeneTex, USA). To evaluate cell viability, serum-free media (0.5 mL) containing 10% AlamarBlue assay reagent was topped up to each well of the 12-well plates. After 2 h at 37 °C, the media (100 μL) were transferred to black-bottomed 96-well plates, and the fluorescence signals were recorded on Spark 10 M microplate reader (Tecan, Switzerland) at 590 nm of emission and at 540 nm of excitation. Data were expressed as percentages of FI of analyzed cells compared to untreated controls.

### Bioluminescence imaging study

All animal procedures were overseen by the Institutional Animal Care and Use Committee (IACUC) of the Singapore Biological Resource Centre (BRC, approved protocol number: 221681). Female BALB/cAnNTac (BALB/c) mice at 5–6 weeks of age were obtained from InVivos Pte Ltd (Singapore) and maintained in a specific pathogen-free facility of BRC. Ten BALB/c mice were inoculated with FLuc mRNA-LNPs containing 1 μg of mRNA in 20 μL via subcutaneous injection in the hind leg area using a 3/10cc insulin syringe (BD, USA). Five naive mice injected with luciferin served as a mock control. At indicated time points, VivoGlo luciferin (Promega, USA) was intraperitoneally injected into the mice at a dose of 150 mg per kg. After 10 min, the IVIS Spectrum imaging system (PerkinElmer, USA) was employed to obtain the luminescence signals of the mice. Organs (spleen, liver, kidney, lung, heart, inguinal lymph node, and skin) were harvested immediately for ex vivo luminescence imaging. The total flux values in each region of interest were measured using the Living Image software (PerkinElmer, USA).

### In vivo vaccination experiments

Twenty female BALB/c mice (InVivos Pte Ltd, 5–6 weeks of age) were immunized with 1 μg of PVX1010 mRNA loaded in LNPs in 20 μL via subcutaneous injection in the hind leg area and boosted with the same dose of PVX1010 mRNA-LNP on day 21 post initial immunization. Eight weeks after the 1st boost, the mice were allocated randomly to 4 different treatment groups (5 mice per group) and then injected with PVX1010, PVX1020, PVX1040, or PVX1050 mRNA-LNPs (mRNA dose = 1 μg/mouse) for the 2nd boost immunization. Blood was collected weekly by tail vein bleeding, followed by centrifugation and serum collection. The mouse body weight was also measured throughout the course of the vaccination study. At the endpoint, the mice were euthanized by CO_2_ inhalation to collect the sera and organs. Serum concentration of BUN, ALT, and LDH was quantified using the corresponding enzymatic assay kit (Bioo Scientific, USA). Five unimmunized mice were used as a negative control. Liver, spleen, and heart tissues fixed with 10% formalin were sectioned at 4 µm and then stained by hematoxylin and eosin (H&E).

### Expression of SARS-CoV-2 RBD antigens and ACE2-Fc

The expression and purification of RBD and ACE2-Fc were done as described in the previous report^44^. RBD containing His-tag was purified *via* the ÄktaPure liquid chromatography system using HisTrap HP His-tag protein purification columns (Cytiva, USA). The constructs for the RBD variants, except for the Omicron BA.1 and BA.2 were produced with KLD enzyme mix (New England Biolabs, USA) using RBD as the template. Omicron BA.1 and BA.2 (I332-E554)-coding genes were produced by Integrated DNA Technologies (Coralville, USA). Original RBD gene coding for the same region was replaced with the respective sequence using Gibson assembly (New England Biolabs, USA). The primers used are disclosed in Supplementary information (Supplementary Table 2). The expression and purification for the RBD variants were performed identically to the method used for RBD.

### ACE2-RBD binding inhibition ELISA

The presence of neutralising antibodies in vaccinated mice sera was evaluated using an ELISA-based surrogate viral neutralisation test^22,23^. The Delta and Wuhan-Hu-1 RBD antigens (1 μg/mL) and the Omicrons BA.1 and BA.2 (4 μg/mL) were coated onto a flat-bottomed 96-well MaxiSorp immunoplate (SPL Life Sciences #32296, South Korea) for 30 min at room temperature. The plate was rinsed thrice using an automated washer (Biotek EL 406) with a washing buffer (0.05% Tween-20-supplemented PBS) and then treated for 60 min with a blocking buffer (the washing buffer supplemented with 3% bovine serum albumin). The serum samples were then diluted 10 times in blocking buffer. In addition, the negative controls (heat-inactivated FBS diluted 5-fold in the blocking buffer) and positive controls (100 μg/mL ACE2-FC in 5× FBS) were prepared for each RBD variant. Following the incubation period, the washing step was repeated. The respective serum samples, negative and positive controls were added onto the plates for 45 min at room temperature. Following the primary incubation, the washing procedure was repeated. The plates were then treated with the HRP-conjugated ACE2-Fc detector antibody at 600 ng/mL for the Omicron variants and at 200 ng/mL for the Wuhan-Hu-H1 and Delta variants, for 45 min at room temperature in the dark. The wash step was repeated, and TMB (3,3’,5,5’-tetramethylbenzidine) substrate (Thermo Scientific #34029, USA) was added. After 3 min of incubation, the reaction was stopped by addition of 1 M H_2_SO_4_ solution. The optical density at 450 nm (OD_450_) was then recorded using a Tecan 100 M microplate reader. The formula below was used to calculate the percentage inhibition values. The threshold was set at 40% blocking based on comparison with the pseudovirus neutralization test in our previous works^22,45^.

$${\rm{Inhibition}}\,( \% )=\frac{{Readout}\,\left({negative}\,{control}\right)-{Readout}\,\left({sample}\right)}{{Readout}\,\left({negative}\,{control}\right)}\times 100$$

### Statistical analysis

All graphs were plotted as mean ± standard deviation (SD). The Prism 8 software (GraphPad Software, USA) was used to conduct two-tailed unpaired Student’s *t* tests for two groups or a one-way analysis of variance with Tukey’s post hoc test for three or more groups. A *p* value below 0.05 was considered to be statistically significant.

### Reporting summary

Further information on research design is available in the Nature Research Reporting Summary linked to this article.

### Supplementary information

Supplementary Information

Reporting Summary

## Data Availability

The data generated and/or analysed in the current study are available from the corresponding authors on reasonable request.
