# Supplementary material for: Durable cross-protective neutralizing antibody responses elicited by lipid nanoparticle-formulated SARS-CoV-2 mRNA vaccines
Source: NPJ Vaccines. 2024 Feb 23;9:43. doi: 10.1038/s41541-024-00835-x (PMC10891077; doi:10.1038/s41541-024-00835-x)
Supplement: Supplementary file 1 — Supplementary Information [file 41541_2024_835_MOESM1_ESM.pdf]

Supplementary information for

Durable cross-protective neutralizing antibody responses elicited by lipid nanoparticle-formulated SARS-CoV-2 mRNA vaccines

Ki Hyun Bae[1,†], Bhuvaneshwari Shunmuganathan[2,3,†], Li Zhang[1,†], Andrew Lim[4], Rashi Gupta[2,3], Yanming Wang[1], Boon Lin Chua[1], Yang Wang[5], Yue Gu[2,3], Xinlei Qian[3], Tan Siang Ling Isabelle[3], Kiren Purushotorman[2,3], Paul A MacAry[2,3,*], Kevin P White[5,6,*], Yi Yan Yang[1,*]

[1]Bioprocessing Technology Institute (BTI), Agency for Science, Technology and Research (A*STAR), 20 Biopolis Way, Centros #06-01, Singapore 138668, Republic of Singapore

[2]Department of Microbiology and Immunology, Yong Loo Lin School of Medicine, National University of Singapore, Singapore 117545, Republic of Singapore

[3]NUS-Cambridge Immune Phenotyping Centre (NCIPC), Life Sciences Institute, National University of Singapore, Singapore 117456, Republic of Singapore

[4]Provaxus, Inc.

[5]Genome Institute of Singapore (GIS), Agency for Science, Technology and Research (A*STAR), 60 Biopolis St, Singapore 138672, Republic of Singapore

[6]Department of Biochemistry and Precision Medicine Translational Research Program, Yong Loo Lin School of Medicine, National University of Singapore, Singapore 119228, Republic of Singapore

[†] These authors contributed to this study equally.

[*]Co-corresponding authors: mdckpw@nus.edu.sg (K.P. White); micpam@nus.edu.sg (P.A. MacAry); yyyang@bti.a-star.edu.sg (Y.Y. Yang)

Supplementary Table 1.

Polydispersity index and encapsulation efficiency of 4 different COVID-19 vaccine mRNA-LNPs.

Formulation	Polydispersity index (PDI)	Encapsulation efficiency (%)
PVX1010 mRNA-LNPs	0.127 ± 0.060	94.3
PVX1020 mRNA-LNPs	0.030 ± 0.003	93.5
PVX1040 mRNA-LNPs	0.077 ± 0.024	90.9
PVX1050 mRNA-LNPs	0.082 ± 0.008	93.7

Supplementary Table 2.

Sequence of the primers used to generate constructs for RBD variants.

Primer	Sequence
D21 R	CAAAAGGGCACAAGTTTGTAATATTAGGAA
I14 F	ATTACAAACTTGTGCCCTTTTGATGAAG
G230 F	GCACAGGTGTTCTTACTGAGTCTAAC
E236 R	CTCAGTAAGAACACCTGTGCCTTTTAA
E236 T R	CTCAGTAAGAACACCTGTGCCTGTTAA

a

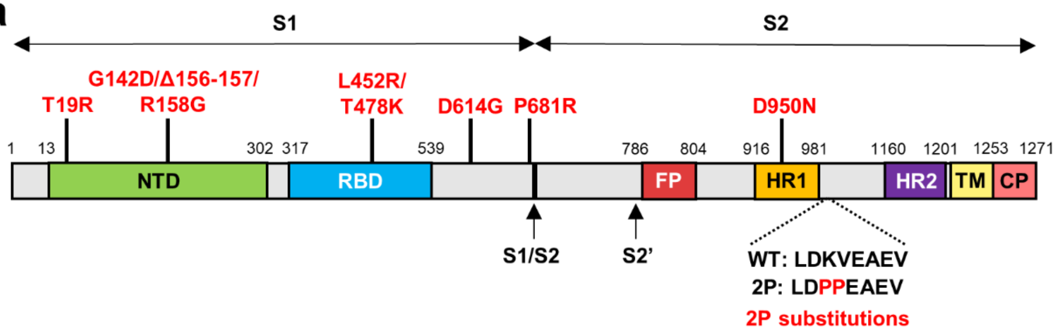

b

```
   1 mfvflvllpl vssqcvnlrt rtqlppaytn sftrgvyypd kvfrssvlhs tqdlflpffs
  61 nvtwfhaihv sgtngtkrfd npvlpfndgv yfasteksni irgwifgttl dsktqslliv
 121 nnatnvvikv cefqfcndpf ldvyyhknnk swmesgvyss anndctfeyvs qpflmdlegk
 181 qgnfknlref vfknidgyfk iyskhtpinl vrdlpqgfsa leplvdlpig initrfqtll
 241 alhrsyltpg dsssgwtaga aayyvgylqp rtfllkynen gtitdavdca ldplsetkct
 301 lksftvekgi yqtsnfrvqp tesivrfpni tnlcpfgevf natrfasvya wnrkrisncv
 361 adysvlynsa sfstfkcygv sptklndlcf tnvyadsfvi rgdevrqiap gqtgkiadyn
 421 yklpddftgc viawnsnnld skvggnynyr yrlfrksnlk pferdistei yqagskpcng
 481 vegfncyfpl qsygfqptng vgyqpyrvvv lsfellhapa tvcgpkkstn lvknkcvnfn
 541 fngltgtgvl tesnkkflpf qqfgrdiadt tdavrdpqtl eilditpcsf ggvsvitpgt
 601 ntsnqvavly qgvnctevpv aihadqltpt wrvystgsnv fqtragclig aehvnnsyec
 661 dipigagica syqtqtnsrr rarsvasqsi iaytmslgae nsvaysnnsi aiptnftisv
 721 tteilpvsmt ktsvdctmyi cgdstecsnl llqygsfctq lnraltgiav eqdkntqevf
 781 aqvkqiyktp pikdfggfnf sqilpdpskp skrsfiedll fnkvtladag fikqygdclg
 841 diaardlica qkfngltvlp plltdemiaq ytsallagti tsgwtfgaga alqipfamqm
 901 ayrfngigvt qnvlyenqkl ianqfnsaig kiqdslssta salgklqnvv nqnaqalntl
 961 vkqlssnfga issvlndils rldppeaevq idrlitgrlq slqtyvtqql iraaeirasa
1021 nlaatkmsec vlgqskrvdf cgkgyhlmsf pqsaphgvvf lhvtyvpaqe knfttapaic
1081 hdgkahfpre gvfvsngthw fvtqrnfyep qiittdntfv sgncdvvigi vnntvydplq
1141 peldsfkeel dkyfknhtsp dvdlgdisgi nasvvniqke idrlnevakn lneslidlqe
1201 lgkyeqyikw pwyiwlgfia gliaivmvti mlccmtsccs clkgccscgs cckfdeddse
1261 pvlkgvklhy t*
```

Supplementary Figure 1. Design of mRNA vaccine encoding the full-length spike glycoprotein of the Delta variant of SARS-CoV-2. **a** Linear diagram of the spike glycoprotein encoded by PVX1010 mRNA. N-terminal domain (NTD), receptor binding domain (RBD), S1/S2 cleavage site (S1/S2), S2 cleavage site (S2′), fusion peptide (FP), heptad repeat 1 (HR1), heptad repeat 2 (HR2), transmembrane domain (TM) and cytoplasmic tail domain (CP). Amino acid mutations, deletions and substitutions are shown in red text. **b** Protein sequence of the spike glycoprotein encoded by PVX1010 mRNA. Amino acid changes are highlighted in pink.

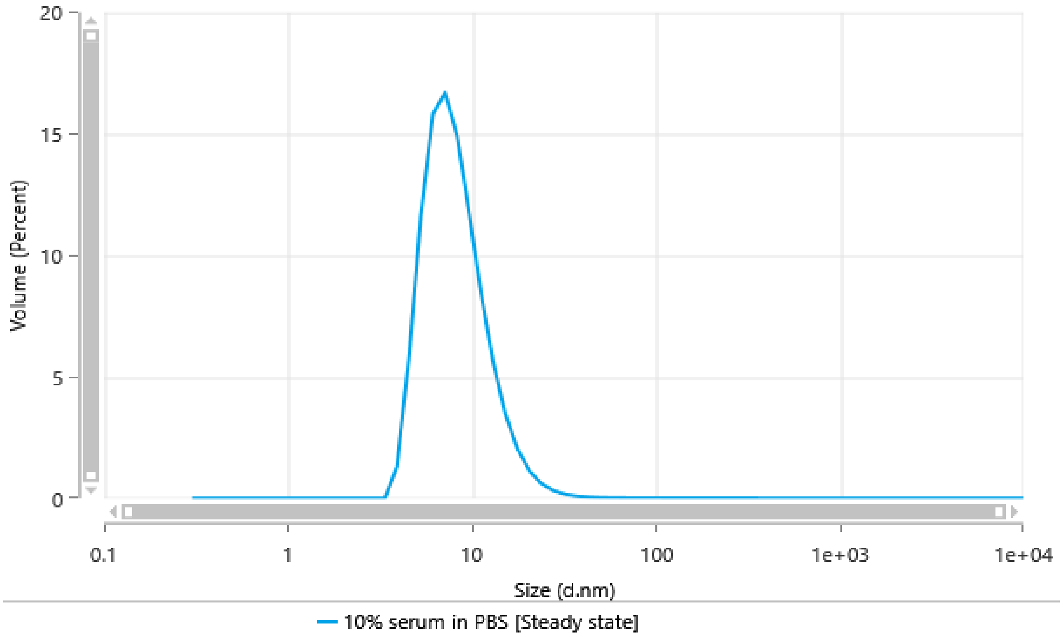

Supplementary Figure 2. Size distribution profile of 10% serum-supplemented PBS.

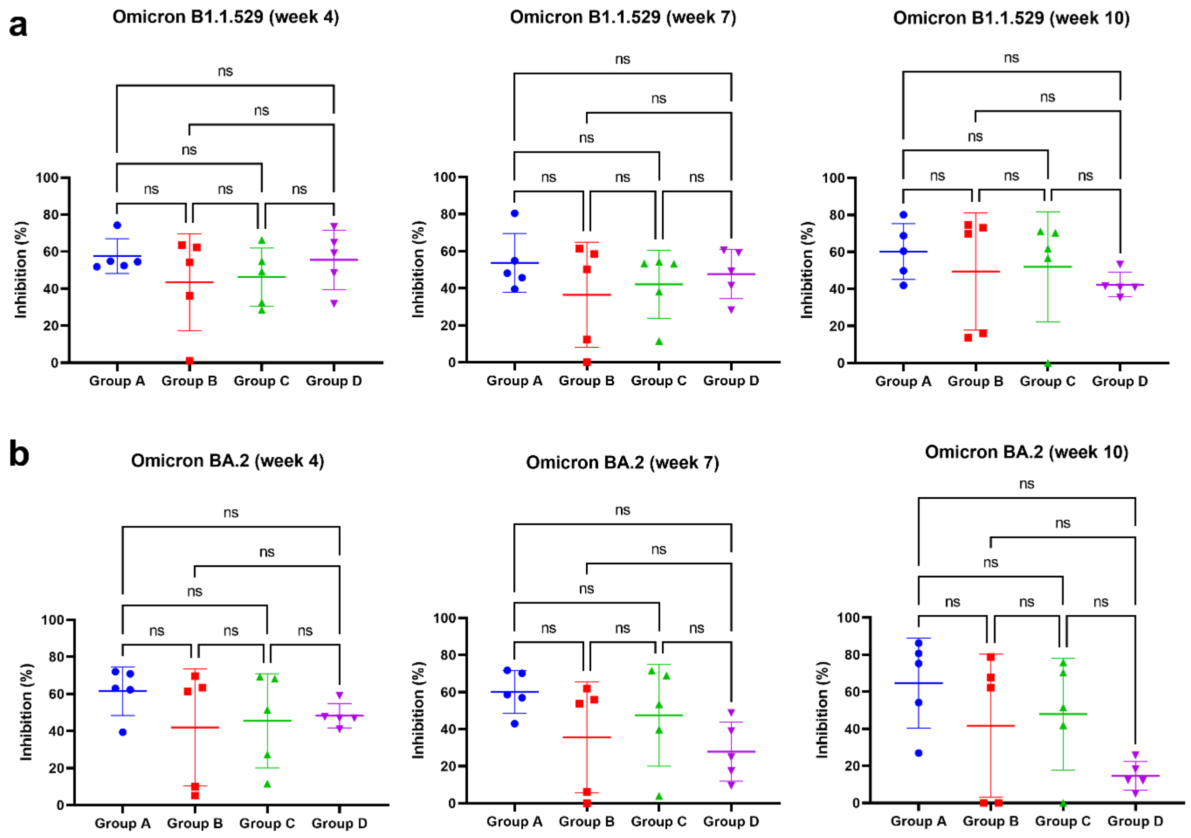

Supplementary Figure 3. ACE2-RBD binding inhibitory response observed for (**a**) Omicron B1.1.529 and (**b**) BA.2 variants at week 4, 7 and 10. The tested vaccination groups include Group A (a three-dose immunization of PVX1010 mRNA-LNPs), Group B (two doses of PVX1010 plus PVX1040 booster), Group C (two doses of PVX1010 plus PVX1050 booster) and Group D (two doses of PVX1010 plus PVX1020 booster). Mean ± SD (n = 5); ns: nonsignificant ($P > 0.05$ from one-way ANOVA with Tukey's post hoc test).

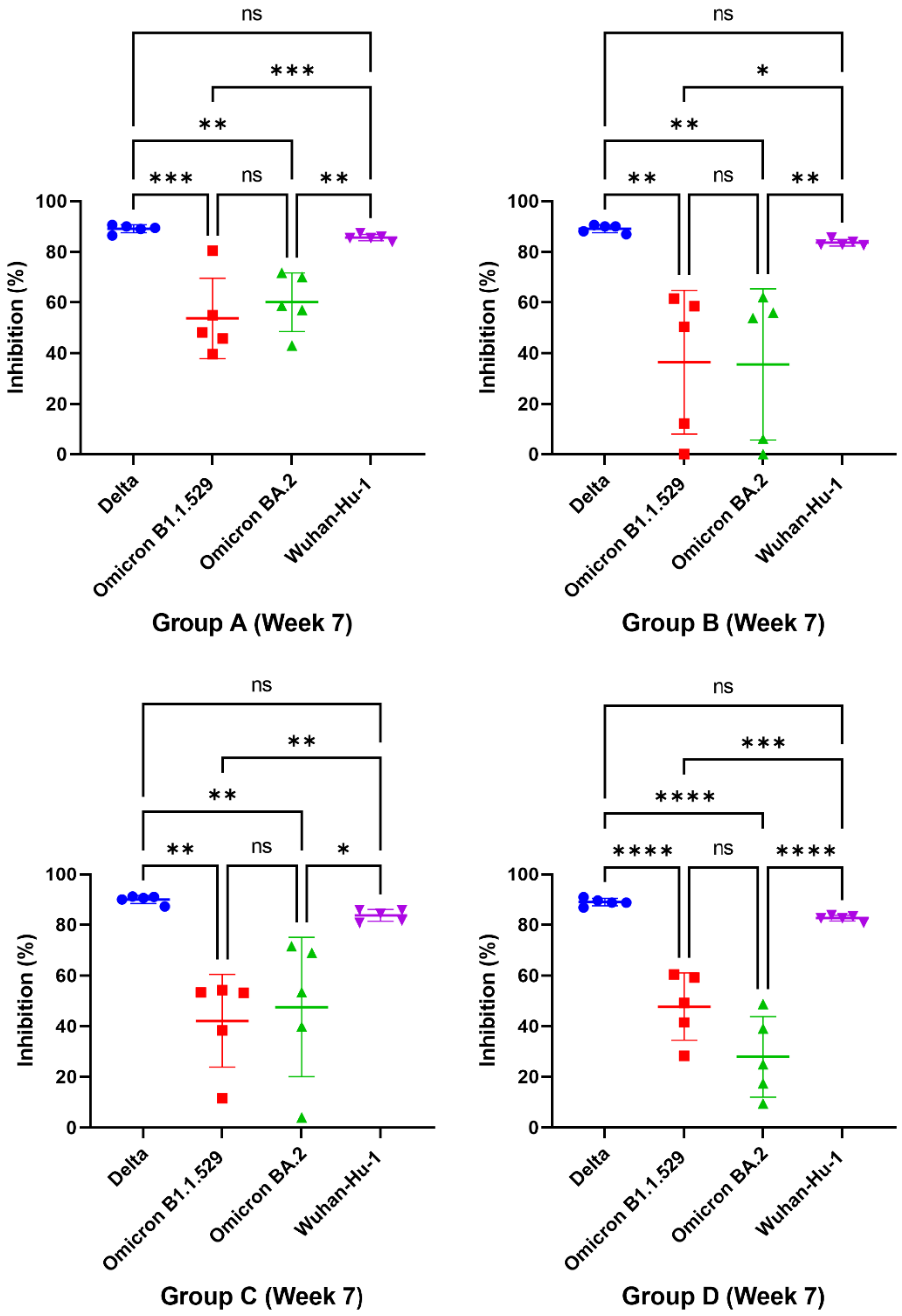

Supplementary Figure 4. ACE2-RBD binding inhibitory responses observed at week 7. The tested vaccination groups include Group A (a three-dose immunization of PVX1010 mRNA-LNPs), Group B (two doses of PVX1010 plus PVX1040 booster), Group C (two doses of PVX1010 plus PVX1050 booster) and Group D (two doses of PVX1010 plus PVX1020 booster). Mean ± SD (n = 5); $^{*}P < 0.05$; $^{**}P < 0.01$; $^{***}P < 0.001$; $^{****}P < 0.0001$; ns: nonsignificant ($P > 0.05$ from one-way ANOVA with Tukey's post hoc test).
