# Supplementary material for: Durable cross-protective neutralizing antibody responses elicited by lipid nanoparticle-formulated SARS-CoV-2 mRNA vaccines
Source: NPJ Vaccines. 2024 Feb 23;9:43. doi: 10.1038/s41541-024-00835-x (PMC10891077; doi:10.1038/s41541-024-00835-x)
Supplement: Supplementary file 2 — Reporting Summary [file 41541_2024_835_MOESM2_ESM.pdf]

nature portfolio

Corresponding author(s): NPJVACCINES-03499

Last updated by author(s): Dec 14, 2023

# Reporting Summary

Nature Portfolio wishes to improve the reproducibility of the work that we publish. This form provides structure for consistency and transparency in reporting. For further information on Nature Portfolio policies, see our Editorial Policies and the Editorial Policy Checklist.

## Statistics

For all statistical analyses, confirm that the following items are present in the figure legend, table legend, main text, or Methods section.

| n/a | Confirmed | |
|---|---|---|
| ☐ | ☒ | The exact sample size (*n*) for each experimental group/condition, given as a discrete number and unit of measurement |
| ☐ | ☒ | A statement on whether measurements were taken from distinct samples or whether the same sample was measured repeatedly |
| ☐ | ☒ | The statistical test(s) used AND whether they are one- or two-sided<br>*Only common tests should be described solely by name; describe more complex techniques in the Methods section.* |
| ☐ | ☒ | A description of all covariates tested |
| ☐ | ☒ | A description of any assumptions or corrections, such as tests of normality and adjustment for multiple comparisons |
| ☐ | ☒ | A full description of the statistical parameters including central tendency (e.g. means) or other basic estimates (e.g. regression coefficient) AND variation (e.g. standard deviation) or associated estimates of uncertainty (e.g. confidence intervals) |
| ☐ | ☒ | For null hypothesis testing, the test statistic (e.g. *F*, *t*, *r*) with confidence intervals, effect sizes, degrees of freedom and *P* value noted<br>*Give P values as exact values whenever suitable.* |
| ☒ | ☐ | For Bayesian analysis, information on the choice of priors and Markov chain Monte Carlo settings |
| ☒ | ☐ | For hierarchical and complex designs, identification of the appropriate level for tests and full reporting of outcomes |
| ☒ | ☐ | Estimates of effect sizes (e.g. Cohen's *d*, Pearson's *r*), indicating how they were calculated |

*Our web collection on statistics for biologists contains articles on many of the points above.*

## Software and code

Policy information about availability of computer code

| | |
|---|---|
| Data collection | All IVIS images were collected using Living Image software (PerkinElmer, USA). |
| Data analysis | All statistical analyses were performed using Prism 8 software (GraphPad Software, USA). |

For manuscripts utilizing custom algorithms or software that are central to the research but not yet described in published literature, software must be made available to editors and reviewers. We strongly encourage code deposition in a community repository (e.g. GitHub). See the Nature Portfolio guidelines for submitting code & software for further information.

## Data

Policy information about availability of data

All manuscripts must include a data availability statement. This statement should provide the following information, where applicable:

- Accession codes, unique identifiers, or web links for publicly available datasets
- A description of any restrictions on data availability
- For clinical datasets or third party data, please ensure that the statement adheres to our policy

The data generated and/or analysed in the current study are available from the corresponding authors on reasonable request.

## Research involving human participants, their data, or biological material

Policy information about studies with human participants or human data. See also policy information about sex, gender (identity/presentation), and sexual orientation and race, ethnicity and racism.

| | |
|---|---|
| Reporting on sex and gender | No human research was done in this study |
| Reporting on race, ethnicity, or other socially relevant groupings | No human research was done in this study |
| Population characteristics | No human research was done in this study |
| Recruitment | No human research was done in this study |
| Ethics oversight | No human research was done in this study |

Note that full information on the approval of the study protocol must also be provided in the manuscript.

# Field-specific reporting

Please select the one below that is the best fit for your research. If you are not sure, read the appropriate sections before making your selection.

☒ Life sciences ☐ Behavioural & social sciences ☐ Ecological, evolutionary & environmental sciences

For a reference copy of the document with all sections, see nature.com/documents/nr-reporting-summary-flat.pdf

# Life sciences study design

All studies must disclose on these points even when the disclosure is negative.

| | |
|---|---|
| Sample size | 5 mice in each group were used. This sample size has been commonly used for animal studies in many published reports. |
| Data exclusions | No data were excluded from the analyses. |
| Replication | All experiments were repeated at least 3 times. The respective N numbers are indicated in figure captions/methods. The results were reliable and reproducible. |
| Randomization | All animal subjects were randomly allocated into experimental groups. |
| Blinding | Investigators were not blinded to group allocation. |

# Reporting for specific materials, systems and methods

We require information from authors about some types of materials, experimental systems and methods used in many studies. Here, indicate whether each material, system or method listed is relevant to your study. If you are not sure if a list item applies to your research, read the appropriate section before selecting a response.

### Materials & experimental systems

| n/a | Involved in the study | |
|---|---|---|
| ☐ | ☒ | Antibodies |
| ☐ | ☒ | Eukaryotic cell lines |
| ☒ | ☐ | Palaeontology and archaeology |
| ☐ | ☒ | Animals and other organisms |
| ☒ | ☐ | Clinical data |
| ☒ | ☐ | Dual use research of concern |
| ☒ | ☐ | Plants |

### Methods

| n/a | Involved in the study | |
|---|---|---|
| ☒ | ☐ | ChIP-seq |
| ☒ | ☐ | Flow cytometry |
| ☒ | ☐ | MRI-based neuroimaging |

## Antibodies

| | |
|---|---|
| Antibodies used | HRP-conjugated ACE2-Fc detector antibody used was our in-house antibody. We had outsourced to ThermoFisher (Waltham, MA, USA) for large-scale production (Lot number: 3315928). |

| | |
|---|---|
| Validation | ThermoFisher (Waltham, MA, USA) has done validation of the antibody for the species and application. |

## Eukaryotic cell lines

Policy information about cell lines and Sex and Gender in Research

| | |
|---|---|
| Cell line source(s) | HEK293 cells (ATCC, USA) |
| Authentication | The cell line used was obtained from a trusted organization without further authentication. |
| Mycoplasma contamination | The cell line used was tested negative for mycoplasma contamination. |
| Commonly misidentified lines (See ICLAC register) | No commonly misidentified cell lines were used in this study. |

## Animals and other research organisms

Policy information about studies involving animals; ARRIVE guidelines recommended for reporting animal research, and Sex and Gender in Research

| | |
|---|---|
| Laboratory animals | Female BALB/cAnNTac (BALB/c) mice at 5-6 weeks of age were obtained from InVivos Pte Ltd (Singapore) |
| Wild animals | This study did not involve wild animals. |
| Reporting on sex | Finding of this study does not apply to only one sex. |
| Field-collected samples | This study did not involve samples collected from the field. |
| Ethics oversight | All animal procedures were overseen by the Institutional Animal Care and Use Committee (IACUC) of the Singapore Biological Resource Centre (BRC, approved protocol number: 221681). |

Note that full information on the approval of the study protocol must also be provided in the manuscript.

## Plants

| | |
|---|---|
| Seed stocks | This study did not involve plants. |
| Novel plant genotypes | This study did not involve plants. |
| Authentication | This study did not involve plants. |
